# Formation and Stability of the Propionitrile:Acetylene Co-Crystal Under Titan-Relevant Conditions

**DOI:** 10.1021/acsearthspacechem.4c00262

**Published:** 2025-01-28

**Authors:** Ellen C. Czaplinski, Tuan H. Vu, Helen Maynard-Casely, Courtney Ennis, Morgan L. Cable, Michael J. Malaska, Robert Hodyss

**Affiliations:** † NASA Jet Propulsion Laboratory, California Institute of Technology, Pasadena, California 91109, United States; ‡ Australian Nuclear Science and Technology Organisation, Kirrawee DC, NSW 2232, Australia; § Department of Chemistry, University of Otago, Dunedin 9054, New Zealand; ∥ MacDiarmid Institute for Advanced Materials and Nanotechnology, Wellington 6140, New Zealand

**Keywords:** co-crystalline, nitrile, hydrocarbon, Titan, Raman spectroscopy, X-ray diffraction, molecular mineral, labyrinth terrain

## Abstract

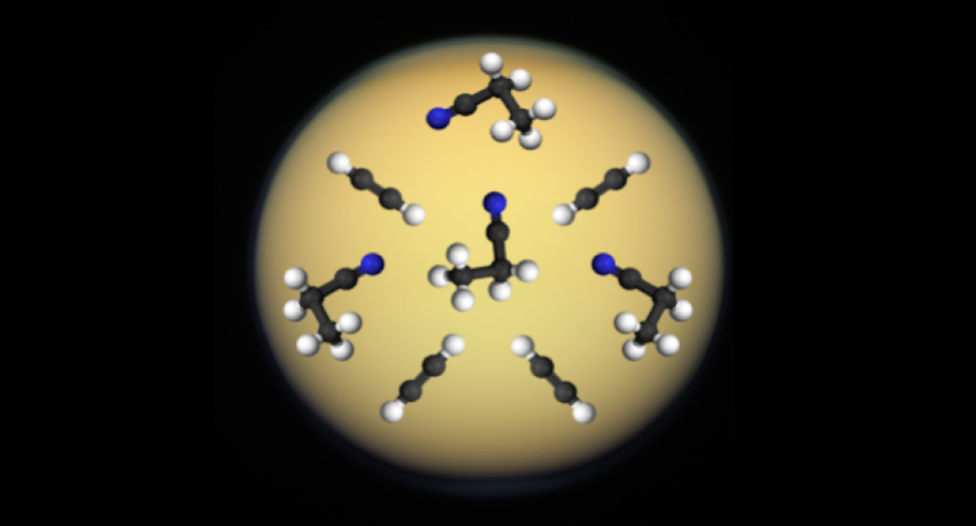

Propionitrile (also known as ethyl cyanide, CH_3_CH_2_CN) and acetylene (C_2_H_2_) are two organic molecules that have been detected in Titan’s atmosphere. Over time, they may interact with each other as they are transported to Titan’s surface. We sought to determine if any reactions or associations such as co-crystal formation might occur between the two molecules. Using micro-Raman spectroscopy, we characterized band shifts, new bands, and morphological changes, which are characteristic of co-crystal formation. We found that the propionitrile:acetylene co-crystal forms within minutes at 90 K and is stable from 90 to 160 K. A cryogenic powder X-ray diffraction study confirms co-crystal formation at 90 K and indexes to a monoclinic unit cell, *P*2_1_/*a*. A thermal expansion study between 90 and 140 K indicates that the co-crystal exhibits anisotropic thermal expansion, with a limited change in the *b* axis over the temperature range. This information gives insight into the preferred form of propionitrile:acetylene and the nature of these molecular interactions under Titan-relevant conditions. We discuss broader implications of the propionitrile:acetylene co-crystal’s participation in forming Titan’s geologic features such as the karstic, labyrinth terrain. Additionally, co-crystals that include acetylene as a coformer may provide a source of energy for acetylenotrophs to harness, should putative life exist on Titan’s surface or in the subsurface. The Dragonfly mission to Titan will explore the nature and distribution of Titan’s organics at the surface; thus, characterizing these organics in the laboratory before surface operations will inform the likely phases Dragonfly may encounter and support data analysis and interpretation of this exciting mission.

## 1 Introduction

Titan, Saturn’s largest moon, is unique within our Solar System, as it has a thick atmosphere dominated by nitrogen and methane. Ultraviolet photons from the Sun (and to a lesser extent, energetic charged particles from Saturn’s magnetosphere) drive chemical transformations via photoionization and electron–ion recombination in the upper atmosphere, leading to the production of larger molecules and aerosols.^1,2^ These aerosols form multiple haze layers, giving Titan its distinct orange appearance at visible wavelengths. Some of these haze particles serve as cloud condensation nuclei, meaning organic molecules may be entrained within droplets of condensable species (methane/ethane) in the atmosphere to create clouds,^3^ and will eventually fall on the surface to be further processed.^4−6^ Photochemically produced compounds delivered to the surface make up the majority of Titan’s exposed surface materials, where they may participate in further processes such as physical/chemical weathering, dissolution, physical erosion, partitioning, deposition, lithification, and possibly remobilization to create the variety of terrains comprising Titan’s surface landscape. Herein, we study the interactions between two observed surface compounds, propionitrile and acetylene, and experimentally determine whether they form an organic co-crystal under Titan-relevant surface conditions. Co-crystals are chemically and structurally distinct from the pure constituents that comprise them, making them important to study to understand present surface conditions and geologic processes that may have occurred in Titan’s past.

Under Titan conditions, certain mixtures of organic molecules have been shown to form co-crystals, which are a type of cryomineral with a specific stoichiometric ratio and are formed via relatively weak intermolecular interactions (e.g., London dispersion forces and pi-bonding). They can form via solid–solid interactions (acetonitrile:acetylene),^7^ liquid–solid interactions (benzene:ethane),^8^ or both (benzene:acetylene)^9,10^ and often have enhanced phase stability as a function of temperature when compared to the pure molecules. In the context of Titan, co-crystals may provide a system that allows compounds to be preferentially concentrated as a molecular mineral.^11,12^ To date, eight Titan-relevant co-crystals have been studied experimentally; they are typically identified by observing spectral shifts in Raman or infrared spectroscopic reflectance bands, as well as structural characterization with X-ray diffraction (XRD) and sample morphology changes with microscopy.^11,13^ Many successfully formed co-crystal systems include acetylene as a co-former, an energy-dense molecule owing to its carbon–carbon triple bond and high energy of formation. Additionally, co-crystals with astrobiologically relevant molecules like acetylene, nitriles, and subsequent derivatives may allow for the concentration of prebiotic ingredients and metabolic energy sources that may facilitate putative life.^13−15^

Propionitrile (CH_3_CH_2_CN; also known as ethyl cyanide) is a simple aliphatic nitrile that is thought to be produced above 900 km^16^ (see Table 1) by the following formation reactions:
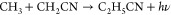

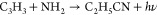


**Table 1:** Formation Reactions of Acetylene and Propionitrile in Titan’s Atmosphere, Density, Altitude in Titan’s Atmosphere, and Mole Fraction

Species	Formula	Formation reaction(s)	Density (g/cm^–3^)	Mole fraction in Titan’s atmosphere	Flux to the Surface (molecules cm^–2^ s^–1^)
acetylene	C_2_H_2_	^3^CH_2_ + ^3^CH_2_ → C_2_H_2_ + H_2_^a^	0.61^c^	3.1 × 10^–4^^d^	5.1 × 10^7^^e^
		C_2_H_4_ + *h*ν → C_2_H_2_ + 2H/H_2_^b^			
propionitrile	CH_3_CH_2_CN	CH_3_ + CH_2_CN → C_2_H_5_CN + *h*ν^f^	1.014^h^	2.8 × 10^–10^^i^	6.4 × 10^6^^j^
		C_3_H_3_ + NH_2_ → C_2_H_5_CN + *h*ν^g^			

a From ref (28).

b From ref (29).

c From ref (30), 194 K.

d From (31), closed source neutral (CSN) mode of Cassini Ion and Neutral Mass Spectrometer (INMS) at 1077 km atmospheric height.

e From refs (32) and (33).

f From ref (34).

g From ref (35).

h From ref (36), 100 K.

i From ref (17).

j From ref (37).

Observations from the Atacama Large Millimeter/submillimeter Array (ALMA) detected propionitrile in Titan’s atmosphere with vertical column densities in the range of (1–5) × 10^14^ cm^–2^, concentrated at altitudes of >200 km, and reported a mole fraction of 2.8 × 10^–10^.^17^ Vuitton et al.^16^ calculated densities of propionitrile at 1.6 × 10^16^ cm^–2^ (between 1000 and 1400 km altitudes), which is about a factor of 50 higher than that reported by Cordiner et al.^17^ In contrast to species like cyanoacetylene (HC_3_N) and acetonitrile (CH_3_CN), the emission maps for propionitrile suggest a seasonally higher abundance in the south polar region than that in the north, consistent with a relatively short chemical lifetime and south polar subsidence.^17^ More recently, Kisiel et al.^18^ conclusively detected vibrationally excited lines of propionitrile in Titan’s atmosphere, marking the first time these transitions have been detected in a planetary body. These measurements allowed for a disk-averaged estimate of 7.78 ± 0.18 ppb (assumed constant above 300 km) for propionitrile.^18^

Acetylene (C_2_H_2_) is a primary photochemical product in Titan’s atmosphere (2.8 × 10^–4^ mole fraction at 1100 km).^16^ The likely formation mechanism of acetylene is a multistep process initiated directly by the photolysis of methane and ethane or indirectly by the photolysis of ethylene (C_2_H_4_) (Table 1). Acetylene has been identified in Titan’s atmosphere and tentatively detected on the surface.^19−23^ Acetylene exists in two distinct crystalline phases as a solid: an orthorhombic low-temperature phase (below 133 K) and a cubic high-temperature phase (133–193 K).^24^ Of these, the orthorhombic phase is expected to be the predominant form on Titan’s surface due to the low surface temperatures (89–94 K).

Propionitrile and acetylene may interact in the atmosphere or on the surface of Titan. According to condensation curves from Yu et al.,^25^ the acetylene gas–solid transition occurs at around 60 km altitude in Titan’s atmosphere and the propionitrile gas–solid transition occurs at around 100 km altitude. Hence, after propionitrile condenses and starts to fall through Titan’s atmosphere, it would be exposed to solid acetylene (among other condensed species) or could cocondense. Surface processes, such as a fluvial event, might also lead to co-crystal formation by introducing an acetylene-saturated methane/ethane flow to a refractory propionitrile surface deposit, as demonstrated with other acetylene-containing co-crystals.^26^

The main objective of this work is to investigate how propionitrile and acetylene behave when mixed under Titan-relevant conditions (90 K, N_2_ atmosphere, 0.1 MPa). We present here the first report of a propionitrile:acetylene co-crystal, and characterize the formation conditions, thermal stability, and proposed structure using the methods described below. The propionitrile:acetylene co-crystal is stable at Titan-relevant temperatures (90 to 160 K) – a temperature range that correlates with Titan’s surface to atmospheric temperatures, as the atmosphere reaches temperatures of >160 K above 100 km altitude.^27^ This study adds to the growing subfield of Titan cryomineralogy studies, helping to further elucidate Titan’s surface-scale composition while informing large-scale geologic processes and interpretation of data from current and future missions.

## 2 Methods

### 2.1 Raman Spectroscopy

Raman spectroscopy has previously been used to identify co-crystal formation, as it provides compositional and environmental context for the molecules being studied. Frequency shifts, sharpening of peaks, and splitting and merging of vibrational modes are typical indicators of co-crystal formation. Raman measurements were performed using a high-resolution confocal dispersive micro-Raman spectrometer (Horiba Jobin-Yvon LabRam HR). Acetylene (Airgas, Inc., industrial grade, dissolved in acetone) was passed through a purifier (Micro Torr MC400–404F, SAES Pure Gas, Inc.) to remove particles of <0.003 μm and organic impurities to <1 pptV (ppt by volume) prior to use, as verified by the absence of Raman spectral features at 787, 1710, and 2922 cm^–1^ corresponding to acetone. After purification, the acetylene was injected into a gas sample bag (0.7 L volume bag constructed of 2 mL Tedlar film, single polypropylene septum fitting, SKC, Inc.) for subsequent deposition.

A 50 μL aliquot of propionitrile (Sigma-Aldrich, ≥99.0%) was deposited onto one of two depressions (or wells) of a 5 mm-thick, two-well microscope slide at 273 K within a liquid nitrogen-cooled optical cryostage (LTS 350, Linkam Scientific Instruments, Ltd.). The propionitrile aliquot was deposited onto the well opposite the liquid nitrogen-cooled area of the stage to allow it to transfer via condensation from the headspace vapor to the lower temperature well of the slide as the temperature decreased. The cryostage was cooled to 263 K and held for ∼3 min before decreasing the temperature in 10 K increments down to 90 K (Titan surface temperature). Acetylene was deposited in a 20 s increment at 90 K. An atmosphere of N_2_ (1 bar, 0.1 MPa) was maintained after acetylene deposition. To facilitate propionitrile and acetylene mixing, the sample was warmed to 160 K, just below the melting point of propionitrile and held for ∼10 s before cooling back to 90 K.

After both compounds were deposited, the sample was observed with the micro-Raman spectrometer through the quartz window of the cryostage, which was mounted onto an XYZ motorized translation stage (Märzhäuser Wetzlar GmbH & Co. KG) underneath the Olympus BXFM objective turret of the micro-Raman spectrometer. The sample was visually observed continuously under various levels of magnification (4×, 10×, 50×) during the experiment. Raman spectra were collected at 0.4 cm^–1^ per pixel resolution using an 1800 grooves/mm grating or 1.7 cm^–1^ resolution using a 600 grooves/mm grating. All samples were excited by a Nd:YAG laser that was frequency-doubled to 532 nm with an output power of 100 mW. The silicon 520.7 cm^–1^ peak was used for frequency calibration. Spectra were collected with acquisition times of 10 to 15 s and 1 accumulation, depending on the signal strength of the particular sample. Thermal stability studies were performed by warming the sample in 10 K increments and obtaining Raman spectra after a 2 min equilibration time at each temperature point. A schematic of the experimental setup for the micro-Raman measurements is depicted in Figure S1.

### 2.2 Powder X-ray Diffraction

Powder XRD is a valuable method for characterizing co-crystal structure, phase, and thermal expansion or contraction. XRD measurements were performed by using a Bruker D8 Discover Da Vinci X-ray diffractometer. To prepare the sample, an ∼8 μL aliquot of propionitrile was deposited into an open-ended borosilicate capillary (0.7 mm internal diameter). The capillary was then mounted and aligned on the goniometer sample attachment of the XRD. A custom-built system for introducing gases (in this case, acetylene), as described in Hodyss et al.^38^ was inserted into the open end of the capillary, which allowed for precise manipulation and deposition of the analyte gas. The system is comprised of two valves and a flowmeter, which are connected to an 8 cm long polyimide-coated silica capillary tube (360 μm outside diameter, 100 μm inside diameter) through a standard 1/8” Swagelok elbow, which is mounted to a manual XYZ micromanipulator.^38^ The silica capillary was slowly directed inside the borosilicate capillary to prepare it for acetylene deposition. Following a nitrogen purge, acetylene gas flow into liquid propionitrile was initiated at room temperature, and the sample temperature was gradually lowered in ∼10 K increments using a liquid nitrogen-cooled Oxford Cryosystems Cryostream 800 (temperature control to within ±1 K) until the mixed sample solidified at 150 K. The sample was then cooled to 90 K. A schematic of the experimental setup for the XRD measurements is depicted in Figure S2.

Co-crystal formation was confirmed immediately after sample solidification via the identification of distinct peaks in the XRD pattern. The injection capillary was subsequently withdrawn, and the borosilicate capillary was rapidly flame-sealed, followed by a wax seal to isolate the sample from the atmosphere during pattern collection. Powder XRD patterns were then collected from 90 to 140 K at intervals of 10 K with 10 min of equilibration at each temperature point (2 s per step with a 2θ angular resolution of 0.02°, which resulted in ∼2 h for each pattern) using a Cu Kα X-ray source (λ = 1.5406 Å) and a linear energy-dispersive LynxEye XE-T 1D detector. All data were analyzed using Bruker’s Diffrac TOPAS suite (version 6). There are inherent chemical hazards involved when working with propionitrile and acetylene; however, no unexpected or unusually high safety hazards were encountered in either the micro-Raman or the XRD experiments.

## 3 Co-Crystal Formation

### 3.1 Vibrational Mode Shifts

**Figure 1:**
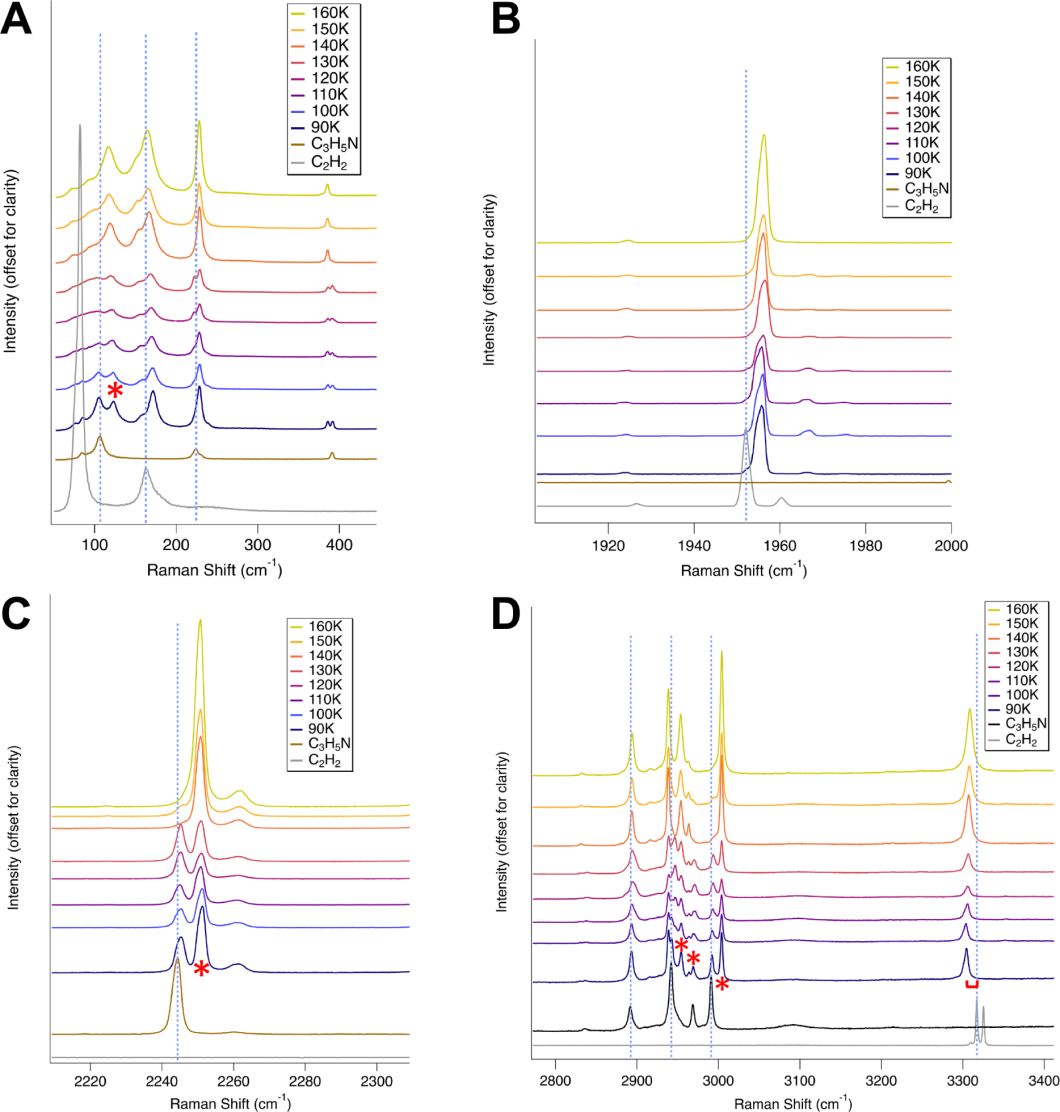
Raman spectra of the propionitrile:acetylene co-crystal from 90 to 160 K, compared to spectra of pure components at 90 K. Spectra are offset for clarity. Lattice modes (A) and C–C stretch of acetylene (B). Vertical dashed lines visually show horizontal offsets compared to vibrational assignments of pure acetylene and propionitrile. Notice the new band at 123 cm^–1^ (denoted by asterisk) and redshifts of bands at 171 cm^–1^, 228 cm^–1^, and 1955 cm^–1^. C≡N stretch (C); CH_2_/CH_3_ stretches of propionitrile; and C–H stretch of acetylene (D). Notice the red shifts and new bands at 2251, 2954, and 2963 cm^–1^ (denoted by asterisks). The acetylene C–H stretch doublet at 3325 cm^–1^ red shifts by ∼13 cm^–1^ and merges into a single peak at 3304 cm^–1^ (D). Note that asterisks are only shown on the 90 K spectrum, although the new bands persist throughout the temperature series.

We compared the Raman spectra of the propionitrile:acetylene mixture with those of the pure components at the same temperature (Figure 1; Tables 2 and 3). After the sample was warmed to 160 K and subsequently cooled to 90 K (see section 2.1.), new bands associated with a change in the molecular environment were observed; this is a common signature of co-crystal formation (e.g., ref (13)). Red shifts and blue shifts up to ∼15 cm^–1^ were observed in several vibrational modes of each molecule (Tables 2 and 3), which are described below.

**Table 2:** Experimental Raman Shifts of Acetylene after co-crystal Formation (90 K) Compared to the Reported Raman Band Centers for Pure Acetylene and the Acetylene Clathrate Hydrate (90 K)

	Raman shift (cm^–1^)				
	Pure acetylene		Acetylene clathrate hydrate	Co-crystal	Δν between pure acetylene and co-crystal^a^
Vibrational mode^b^	Reported^b^ (30 K)	This work (90 K)	Reported^c^ (90 K)	This work (90 K)	This work (90 K)
ν_1_ (C–H stretch)	3314.5	3317		3304.3	–12.7
	3323.5	3325.1			
Water ice (bonded O–H stretch)			3089.3	--	--
ν_2_ (C≡C stretch)	1951.5	1951.8		1955.7	3.9
	1960.5	1960.3	1965.5	1966.2	5.9
			1974.4	--	--
^13^C≡C stretch	1926.5	1926.3		1924.1	–2.2
ν_4_ (C≡C—H bend)	628.5	626.7		--	--
	638.5	636.5		--	--
	659.5	654.5		658.6	4.1
Lattice (vibrations)	84	84.3	81.9	76.5	–7.8
	--	--		85.4	

a Positive value of Δν indicates a blue shift; a negative value indicates a red shift.

b From ref (39) (30 K orthorhombic).

c From ref (40) (90 K).

**Table 3:** Experimental Raman Shifts of Propionitrile after Co-crystal Formation Compared to Reported Raman Band Centers for Pure Propionitrile

	Raman shift (cm^–1^)			
	Pure propionitrile		Co-crystal	Δν between pure and co-crystal
Vibrational mode^b^	Reported^b^ (∼83 K)	This work (90 K)	This work (90 K)	This work^a^
ν_1_ CH_3_ antisymmetric stretch^c^	3006	--	3003.9	--
ν_1_ CH_3_ antisymmetric stretch	2991	2991.1	2991.9	0.8
ν_2_ CH_2_ antisymmetric stretch	2967	2968.8	2968.8	--
			2963.7	–5.1
			2954.3	–14.5
ν_3_ CH_2_ symmetric stretch	2941	2941.9	2942.2	0.3
			2938.8	–3.1
c2ν16 in Fermi Resonance with ν2CH_2_ symmetric stretch	2892	2891.7	2893.1	1.4
ν_4_ C≡N stretch/C–C(N) stretch		2259.9	2261.2	1.3
	2244	2244.4	2251.2	6.8
			2245.3	0.9
ν_5_ CH_3_ antisymmetric deformation	1461	1461.1	1463.2	2.1
ν_6_ CH_3_ antisymmetric deformation CH_2_ scissor	1420	1420.1	1424.3	4.2
ν_7_ CH_2_ deformation CH_3_ symmetric deformation	1390	1391.1	1391	–0.1
ν_8_ CH_2_ wag CH_3_ symmetric deformation C–C (ethyl) stretch	1318	1315.8	1316.2	0.4
ν_17_ CH_2_ twist	1264	1265.3	1265.3	--
C–C (ethyl) stretch CH_3_ rock/C–C–C bend	1076	1075.6	1077.8	2.2
ν_10_ C–C (ethyl) stretch C–C(N) stretchCH_3_ rock	1010	1007.8	999.8	–8.0
ν_11_ CH_3_ rock C–C(N) stretch C–C-C bend	840	840.9	839.9	–1.0
ν_19_ CH_2_ rock CH_3_ rockCH_2_ twist	781	781.7	782.1	0.4
ν_12_ C–C–C bend C–C (ethyl) stretch C–C(N) stretch C–C-N in-plane bend CH_2_ wag	545	545.4	545.9	0.5
			552.3	6.9
ν_20_ C–C–N out-of-plane bend torsion	391	391.1	391.7	0.6
			385.7	–5.4
ν_13_ C–C–N in-plane bend C–C–C bend	224	223.9	228.5	4.62
Lattice modes—unassigned	106.3		105.3	–1
			123.0	16.7

a Positive value of Δν indicates a blue shift; a negative value indicates a red shift.

b From ref (41).

c From ref (42).

Low-frequency rotational and translational motions of molecules in the solid phase define the lattice vibrations (∼50–200 cm^–1^). New lattice modes were observed at 76.5, 85.4, 171.8, and 123.0 cm^–1^. The C–C–N out-of-plane bend mode of propionitrile (ν_20_: 390 cm^–1^) split into two distinct bands upon co-crystal formation (Figure 1A).

The acetylene C≡C stretch bands (ν_2_: 1951.8/1960.3 cm^–1^) are associated with changes in the distance between the acetylene C atoms as the bond stretches and compresses. The acetylene ν_2_ mode red shifts by 3.9 and 5.9 cm^–1^ for the two associated bands (Figure 1B). These shifts in the acetylene bands are distinct from those in the acetylene clathrate hydrate bands (Table 2). A shoulder on the left slope of 1951.8 cm^–1^ is also observed from 90 to 130 K.

The C≡N stretch of propionitrile (ν_4_: 2259.9/2244.4 cm^–1^) is associated with stretching of the triple bond between the C and N nuclei in the nitrile functional group. In the co-crystal spectra, these bands split and exhibit red shifts up to 6.8 cm^–1^ in the co-crystal spectrum (Figure 1C). In the transition from the 130 to 140 K spectrum, a clear change in the shape and intensity of the peaks is observed. In the spectra from 140 to 160 K, the 2245.3 cm^–1^ band decreases in intensity and becomes a shoulder on the left slope of the 2250.8 cm^–1^ band (shifted from 2251.2 cm^–1^ at 90 K).

The CH_2_/CH_3_ symmetric and antisymmetric stretches of propionitrile (ν_1_, ν_2_, and ν_3_) are located in the 2991.1–2941.9 cm^–1^ region. After co-crystal formation, several new bands form, splitting occurs, and red shifts are observed up to 12.8 cm^–1^ (Figure 1 D).

The C–H stretching mode of acetylene (ν_1_: ∼ 3300–3350 cm^–1^) splits from two sharp peaks at 3317 and 3325.1 cm^–1^ to one broader peak at 3304 cm^–1^, a blue shift of 12.7 cm^–1^ (Figure 1D). Similar to previously reported co-crystals, a blue shift and transition to a single peak for this vibrational mode is an indicator of co-crystal formation, due to changes in hydrogen bonding or other intermolecular associations upon co-crystal formation.^7,12^

### 3.2 Sample Morphology

**Figure 2:**
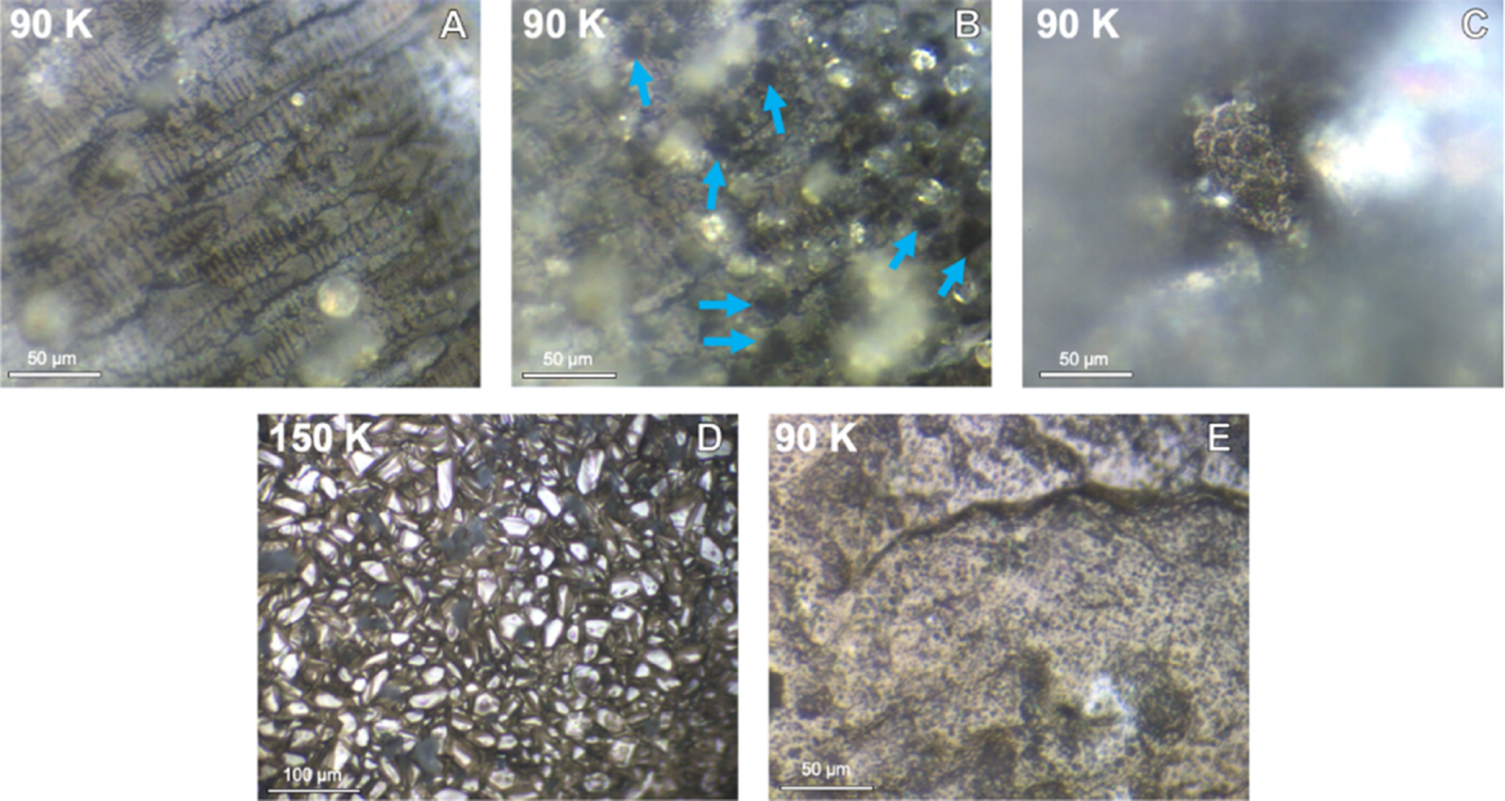
A series of images showing how the sample morphology evolves throughout co-crystal formation. Pure propionitrile at 90 K (a) and propionitrile after acetylene deposition at 90 K with arrows denoting examples of relatively dark acetylene crystals (b). The composition of these dark crystals was confirmed to be acetylene and is shown in Figure S3. Combined sample at 90 K (c), combined sample just below the melting point at ∼150 K (d), and a propionitrile:acetylene co-crystal after recooling to 90 K.

Pure crystallized propionitrile is shown in Figure 2a. Crystallized acetylene can be seen as dark circles in Figure 2b (several examples are denoted by arrows). Raman spectra acquired before and after acetylene deposition confirm that the composition of these darker parts of the sample is indeed pure acetylene (Figure S3). After acetylene condensation, the sample was cooled to 90 K, giving the slightly amorphous morphology seen in Figure 2c. To replicate interaction at warmer temperatures in Titan’s atmosphere, the sample was warmed to ∼160 K (∼200 km altitude),^1^ just below the melting point of the co-crystal sample. Large (25–50 μm) individual crystals formed during this warming period, as can be seen in Figure 2d. The sample was then rapidly cooled to 90 K, resulting in the texture shown in Figure 2e.

## 4 Co-Crystal Structural Characterization: X-ray Diffraction, Thermal Stability, and Expansion

**Figure 3:**
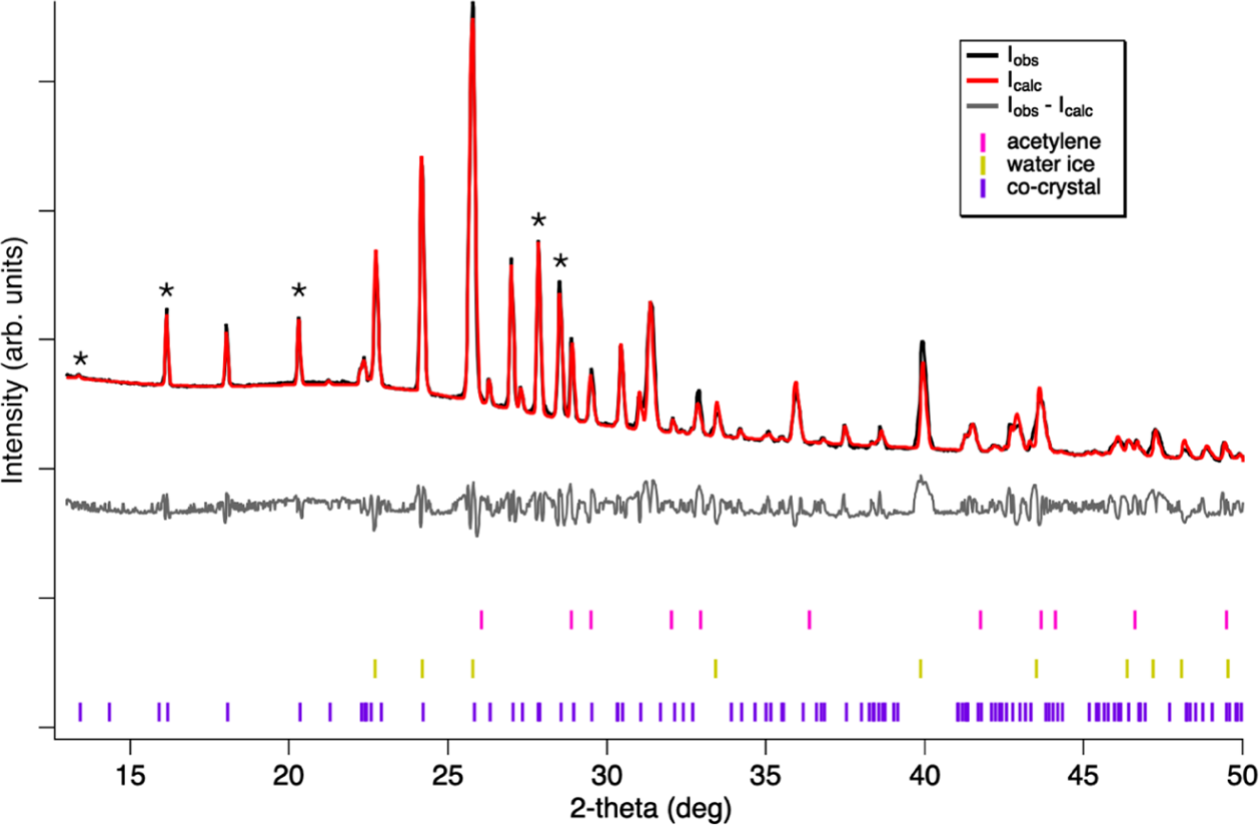
XRD pattern at 90 K (black) which was determined to contain the novel co-crystal, residual acetylene from the formation, and water ice from the outside of the capillary. Also plotted is the result of the modeled pattern (red) and residual pattern (gray, offset for clarity). Tick marks below the patterns represent the Bragg peak positions of acetylene (magenta), water ice (gold), and the co-crystal (purple). The co-crystal is most noticeable by diffraction peaks at 13.38, 16.14, 20.29, 27.84, and 28.50 2θ° (denoted by asterisks).

The propionitrile:acetylene co-crystal forms within minutes at 90 K. Raman spectra indicate thermal stability from Titan surface temperatures (∼90–95 K) to 160 K, above which the co-crystal dissociates. The obtained XRD pattern at 90 K is shown in Figure 3 and was determined to include solid acetylene, water ice (from the outside of the capillary), and a novel phase assumed to be the co-crystal. Excluding the peaks from the acetylene and water ice, the resulting peaks were indexed to a monoclinic unit cell: *a =* 8.85(5) Å, *b =* 9.816(5) Å, *c =* 7.367(7) Å, and β = 116.492(3)°, resulting in a volume of 573.1(6) Å^3^. Systematic absences indicate that the structure is likely to be described by *P*2_1_/*a* symmetry, and with a general position of 4; hence, a 1:1 co-crystal would give a density of 0.935 g/cm^3^ at 90 K. Structure solution is in progress, but the mix-phased nature of the patterns has meant that this has proved challenging to date.

**Figure 4:**
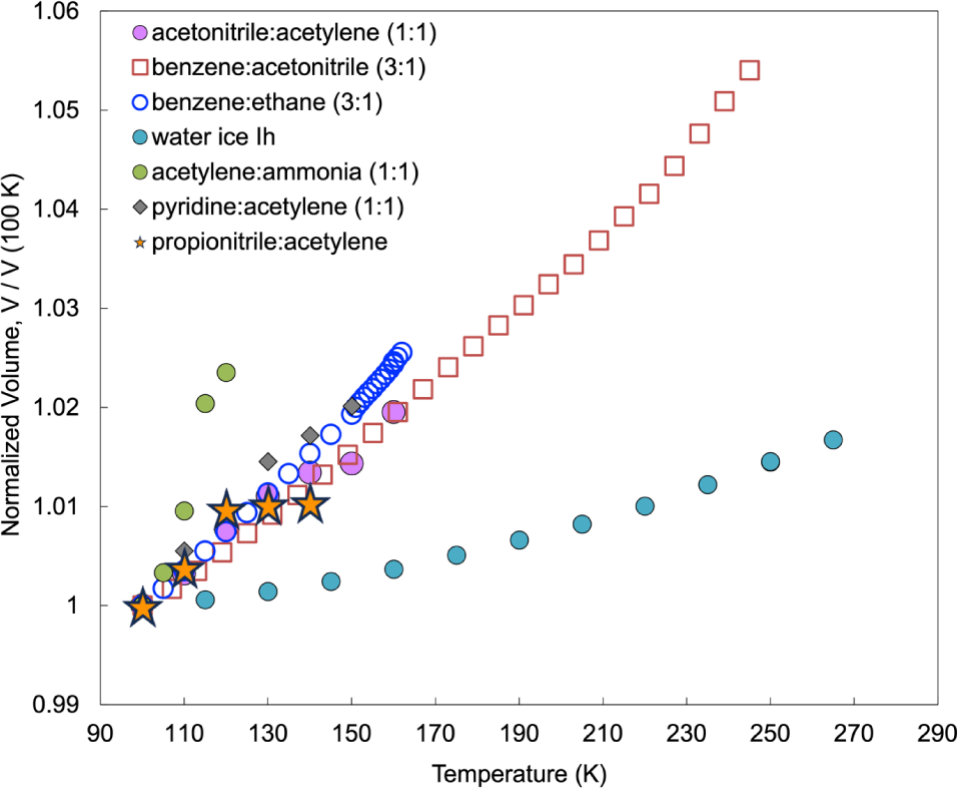
Thermal expansion as a function of normalized volume (at 100 K) for six Titan-relevant co-crystals, as compared to water ice Ih. The propionitrile:acetylene co-crystal volume expansion is shown represented by orange stars (graphic adapted from Cable et al.^13^). Note how the thermal expansion has two regimes: an upward slope from 100 to 120 K, followed by a flat region from 120 to 140 K.

The co-crystal was studied as a function of temperature from 90 to 140 K (the 90 K pattern and the resulting Rietveld refinement are shown in Figure 3; the temperature series is presented in Figure S4). Diffraction peaks at 13.38, 16.14, 20.29, 27.84, and 28.50 θ° are distinct from the pure components and are immediately identifiable after the propionitrile–acetylene mixture solidified at 150 K (freezing point depression) within the XRD capillary. At each temperature step, the diffraction pattern was modeled with the following scheme: peak shape and sample displacement were refined from the first pattern and fixed for subsequent refinements; then, background and crystallite size for each phase were allowed to refine, as well as a Pawley refinement of the co-crystal, and Rietveld refinements (with preferred orientation) to describe the acetylene and water ice in the pattern. Table S1 lists the refined lattice constants and unit cell volume of the propionitrile:acetylene co-crystal from 90 to 140 K, and these values are plotted as a function of temperature to show the thermal expansion of the co-crystal from 90 to 140 K in Figure S5.

## 5 Stability after the Ethane Wetting Event

**Figure 5:**
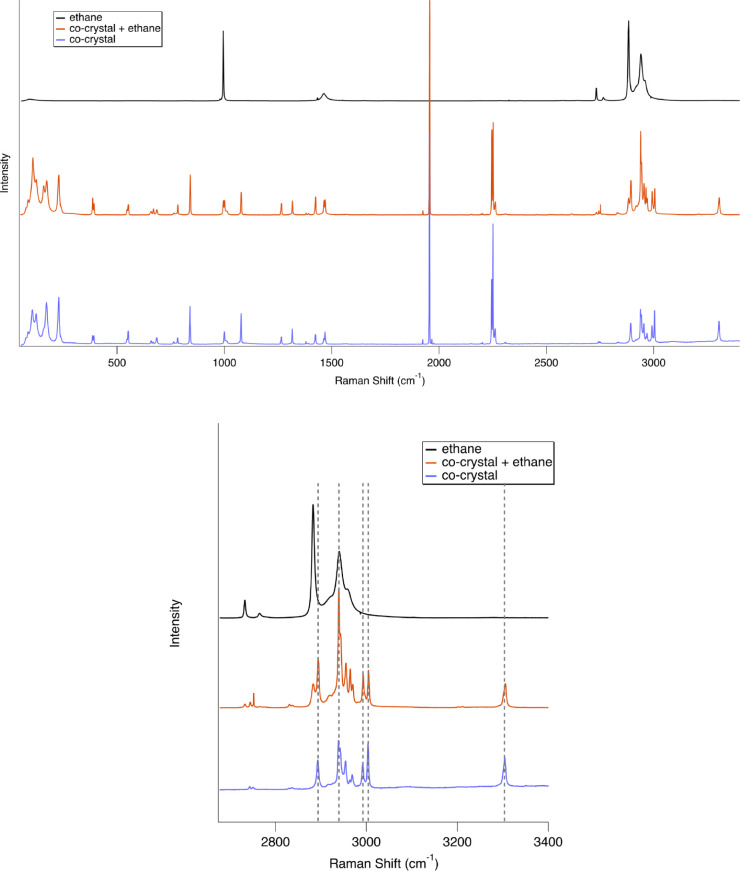
Raman spectra of liquid ethane (black) compared to the co-crystal + ethane (red) and the co-crystal before ethane exposure (blue) (all at 90 K). Spectra are offset for clarity. The full spectrum is shown (top), as well as a zoomed-in region of the CH_2_/CH_3_ stretches of propionitrile and C–H stretch of acetylene (bottom). The presence of ethane bands along with preservation of diagnostic co-crystal features confirm that the co-crystal is stable after short-term exposure to liquid ethane. Dashed lines visually show the lack of change in co-crystal bands between the co-crystal spectrum (blue) and the co-crystal + ethane spectrum (red).

When the Huygens probe landed on Titan’s surface, it detected evidence of volatilized ethane at its landing site^43,44^ due to the heat from the probe, providing support for liquid ethane on Titan. Additionally, clouds and rainstorms have been observed on multiple occasions.^45−52^ Because liquid ethane and methane have the potential to directly alter Titan’s geology and chemistry (either via rain or as reservoirs in the porous regolith that allow wetting from subsurface liquid slowly concentrating upward), we condensed liquid ethane on top of the propionitrile:acetylene co-crystal in the Raman experiments at 90 K to determine how the stability of the co-crystal may be affected. To simulate an ethane wetting event, liquid ethane was condensed inside the cryostage after co-crystal formation was verified (refer to methods in Section 2.1). Figure 5 shows spectra of the ethane wetting experiment in red (90 K), compared to the co-crystal pattern prior to ethane exposure (90 K; blue). Note that the characteristic co-crystal signatures (e.g., shifts and merging of the acetylene C–H band at 3307 cm^–1^; additional CH_2_/CH_3_ stretching bands around 3000 cm^–1^) are still clearly observable immediately after ethane exposure. Due to limitations of this experimental setup, we were unable to test the long-term stability of the ethane-exposed sample; however, a similar ethane wetting experiment performed on the pyridine:acetylene co-crystal indicated stability over the course of a >20 h experiment.^12^ These results suggest stability over greater-than-experimental time scales, but further analysis is beyond the scope of this specific study.

## 6 Discussion

### 6.1 Comparison with Previously Reported Co-Crystals

We compared co-crystal Raman band shifts of the propionitrile:acetylene co-crystal to other previously reported co-crystals containing acetylene as a co-former (acetonitrile:acetylene, pyridine:acetylene, acetylene:ammonia, and butane:acetylene). For acetonitrile:acetylene, the ν_2_ C≡C stretch bands were blueshifted by 5.3 and 6.0 cm^–1^, which is similar to propionitrile:acetylene blueshifts of 3.9 and 5.9 cm^–1^, respectively. The pyridine:acetylene and acetylene:ammonia ν_1_ C–H stretches red-shifted by −9.9 cm^–1^ and −15.5 cm^–1^, respectively, compared to the propionitrile:acetylene redshift of −12.7 cm^–1^. The butane:acetylene ν_2_ C≡C stretch blueshifted by 12 cm^–1^ compared to a 5.9 cm^–1^ shift for propionitrile:acetylene. The shifts of the propionitrile:acetylene co-crystal are mostly similar in both magnitude and direction to those of the acetonitrile:acetylene co-crystal. This is not unexpected, as both cryominerals contain a polar nitrile molecule interacting with a nonpolar acetylene molecule, so similar intermolecular interactions between the C–N and C–H moieties should lead to comparable shifts in the Raman spectra of these vibrational modes.

When comparing the volumetric thermal expansion of the propionitrile:acetylene co-crystal to other previously reported co-crystals (Figure 4), the slopes from 90 to 120 K match most similarly to other nitrile:acetylene co-crystals (acetonitrile:acetylene (1:2) and pyridine:acetylene (1:1)). This may be due to the similarity in volume and atomic behavior in their crystal lattices. Additionally, the change in slope trend of the volumetric thermal expansion (Figure 4) coincides with the phase change of the acetylene from orthorhombic to cubic, which likely drives this change (i.e., the larger volume in the cubic phase puts strain on the co-crystal in the capillary). This also conveniently demonstrates that the water ice is not *inside* the capillary. Further, the acetonitrile:acetylene co-crystal is stable up to only 120 K in the Raman cryostage setup (but 170 K in the cryogenic XRD capillary–equivalent setup as reported herein),^7^ compared to the propionitrile:acetylene co-crystal, which was stable up to 160 K in Raman experiments and up to ∼150 K in XRD experiments.

We note that the starting ratio of the mixture does not affect the ability to form co-crystals, since they only take on specific stoichiometries regardless (e.g., 1:1, 1:2, and 1:3). It is common to observe an excess of one pure component alongside the co-crystal component. Additionally, only one stoichiometry is identified after varying pure component ratios and temperature in laboratory experiments, meaning even if C_2_H_2_ is in excess on Titan, only the single co-crystal structure is expected.

### 6.2 Relevance to Titan’s Geology

At Titan’s surface, propionitrile and acetylene would both exist in the solid phase.^53^ Acetylene is highly soluble in Titan liquids (methane and ethane),^54^ making it a relatively mobile surface material that may be prone to hydrological transport with respect to geological cycles on Titan. On the other hand, polar nitriles such as propionitrile are poorly soluble in Titan’s nonpolar liquids^55−57^ and may only be transported as either eolian or fluvial suspended clastic materials. Due to their poor solubility, they may exist as lag deposits in river deltas. Thermodynamic modeling results by Stevenson et al.^56^ indicate that when compared to other nitriles (acetonitrile, acrylonitrile, and hydrogen cyanide), propionitrile may be one of the more soluble nitrogen-containing species in Titan-relevant liquids; however, the solubility of nitriles is poorly modeled, and there is a large variation of solubility among species. The demonstrated stability of the propionitrile:acetylene co-crystal after exposure to liquid ethane suggests that this material could be a lag deposit in ethane solvents.

Because the co-crystal stays intact after liquid ethane exposure, one can imagine a scenario where the co-crystal forms at the surface (or in the atmosphere via solid–solid interactions), is transported fluvially into a hydrocarbon lake or sea, and then sinks to the bottom since its density (0.935 g/cm^3^) is greater than that of liquid methane and ethane (∼0.51 g/cm^3^ and ∼0.60 g/cm^3^, for an equilibrium mixture of nitrogen and methane/ethane at 91 K, respectively^58^). If we assume this co-crystal (and others that exhibit stability after exposure to liquid ethane)^7,12,26,59^ sinks to the bottom of Titan’s mare, then we can also assume that co-crystals (and other dense refractory organics such as tholin-like materials) would make up the bottom “organic sludge” layers of a lakebed (wet or dried).^60^ This lake-bottom distribution could be another way to concentrate co-crystals and thus would make for an interesting stratigraphic sequencing sample for in situ missions like Dragonfly, if paleolake or other evaporitic terrain were encountered (e.g., ref (61)). However, we note that on Titan, ternary systems (or greater) of organic co-crystals are likely to exist (e.g., ref (62)), which would increase the complexity of this stratigraphic sequence and therefore its interpretation.

It is also important to consider atmospheric interactions. Although propionitrile and acetylene condense at different altitudes in Titan’s atmosphere (∼64 km for acetylene and ∼102 km for propionitrile; mean altitude, after Yu et al.^25^), as propionitrile descends in the atmosphere, acetylene could condense on top of propionitrile particles, allowing for atmospheric interactions. It is important to note that other species would also condense between the point when propionitrile condenses and when acetylene condenses in this atmospheric scenario.

Co-crystals may be discernible in Titan surface materials with an in situ mission like Dragonfly. This could be done in multiple ways: 1) in DraMS, especially for co-crystals that are stable above the carousel storage temperature of 165 K (butane:acetylene, acetonitrile:acetylene, and benzene:acetonitrile), we may observe the more volatile components when we would not expect to observe them, since they would be stable at higher temperatures in the co-crystal phase. 2) With the microscale imager on DragonCam, morphological differences may be identifiable among “pure” or mixed parts of the surface and co-crystalline/cryominerals on the surface. Previous Titan-relevant co-crystal studies have noted significant morphology changes to the sample after co-crystal formation,^9,12,63^ so this could be another line of evidence in a scenario where a sample cannot be taken but an image could be. 3) The Dragonfly Gamma-ray and Neutron Spectrometer (DraGNS) could also provide indirect evidence of a pure co-crystal deposit via specific elemental abundances consistent with the set stoichiometric ratio of that cryomineral. If co-crystal identification turns out to be impractical with Dragonfly, a future in situ mission to Titan with a payload that included an XRD (e.g., heritage from CheMin^64^ or PIXL^65^) and/or a vibrational spectrometer (e.g., ChemCam^66^ or SHERLOC^67^) would be enabling for co-crystal identification and characterization in Titan surface materials. Such measurements could be made either in contact with surface materials (e.g., an attenuated total reflectance or ATR probe) or at a close (∼1 cm, PIXL) or far (CheMin) stand-off distance. Importantly, targeting an area with both active lakes and dry lakebeds would provide a complementary data set to the dunes, interdunes, and impact crater measurements of Dragonfly.

Comparisons to other nitrile:acetylene co-crystals, such as the acetonitrile:acetylene co-crystal,^7^ can also be made. As with the acetonitrile:acetylene co-crystal, the propionitrile:acetylene co-crystal is also composed of one more-soluble and one less-soluble species. Titan’s labyrinth terrain, for example, exhibits karst-like signatures that could be explained by the deposition of mixed organic molecules, dissolution of soluble materials, and mobilization of less-soluble species.^7^ Geologic features such as the labyrinth terrain may have been formed from a *combination* of soluble/insoluble surface materials (i.e., multiple organic minerals/co-crystals simultaneously). The stability of these co-crystals in ethane solution could serve as a way for formerly soluble materials to become effectively indurated during co-crystal formation.

### 6.3 Astrobiological Implications

Co-crystals that include acetylene as a co-former have the potential to provide an energy source that putative life could utilize.^13^ Acetylenotrophy, the metabolic process where an organism degrades acetylene and utilizes it as a sole carbon and energy source,^68^ has recently been assessed as a viable catabolic pathway that could occur on Titan.^69^ Specifically, this study found that acetylenotrophy energy yields are 75–82 kJ/mol C under simulated Titan ocean conditions (energy yields for methanogenesis, by comparison, vary from 75 to 113 kJ/mol C for the same conditions).^69^ Acetylenotrophy could result in similar cell densities as methanogenesis, making it a viable catabolism process on Titan if microbes are present in the subsurface ocean or in near-surface reservoirs (such as melt pools from meteorite impacts). Because acetylene is highly soluble in methane and ethane, it could be present in Titan’s putative “alkanofers” (subsurface reservoirs akin to aquifers but with liquid alkanes), although life could also exist in the ice shell or in impact melts on the surface.^70^ Based on the diversity of Titan-relevant co-crystals discovered to date, many materials could be available and concentrated in solid form as co-crystals and survive on the order of geologic time scales on the surface or in the interior of Titan. Only by characterizing the full cryomineralogy potential of Titan we can more deeply understand the potential spectrum of habitable environments in this alien world.

## 7 Conclusions

Herein, we demonstrated that the propionitrile:acetylene co-crystal forms within minutes at 90 K, a Titan-relevant surface temperature. Micro-Raman spectra and XRD data indicate thermal stability from 90 to 160 K, relevant to Titan’s surface and atmospheric temperatures. The co-crystal is robust after exposure to an ethane wetting event that simulated liquid interactions likely to occur on Titan’s surface. A monoclinic crystal lattice with a 1:1 ratio of propionitrile:acetylene and a density of 0.935 g/cm^3^ is inferred for the co-crystal. Anisotropic thermal expansivity is observed over the temperature range of 90 to 140 K.

This co-crystal exhibits similarities to the acetonitrile:acetylene co-crystal, such as similar bandshifts and degree of volumetric expansion. Titan’s uplifted and highly dissected labyrinth terrains are formed from materials that are likely indurated sedimentary materials. The process for induration is not known, but the stability of the propionitrile:acetylene co-crystal could serve as a way to stabilize formerly soluble materials. In addition, co-crystals that include acetylene as a co-former may provide a source of energy for acetylenotrophs to harness, should putative life exist on Titan’s surface or in the subsurface. Specifically, the metabolic process of acetylenotrophy may provide energy yields on par with those measured from methanogenesis under Titan ocean conditions.

While the field of organic co-crystals has been established in the past decade, there is still abundant work to be done toward classifying Titan’s cryomineralogical tapestry as a whole. This includes ways to more accurately identify likely co-crystal combinations with different laboratory methods. For example, Hines et al.^71^ outline an experimental setup that records the vapor pressures of compounds in a cryogenic chamber – a considerably efficient way of quickly testing which candidate compounds may be more likely to form a co-crystal. Additionally, quantum mechanical modeling of how known organic co-crystals behave at Titan temperatures (and below) can help predict viable co-crystal combinations.^72^ Ultimately, landed missions to Titan’s surface such as Dragonfly will give us the best clues as to Titan’s compositional story. Until Dragonfly lands, though, we can continue to prepare by performing additional experimental studies on Titan’s cryomineralogy here on Earth.

## Data Availability

Data on the propionitrile:acetylene co-crystal, including Raman spectra and XRD patterns, can be found at https://doi.org/10.48577/jpl.YQQJHZ.

## Supporting Information Available

Schematic diagrams of the Raman and XRD experimental setups (Figures S1 and S2); Raman spectra comparing pure propionitrile to propionitrile + acetylene (Figure S3); a table of the refined lattice constants (Table S1); thermodiffractograms of the co-crystal from XRD experiments (Figure S4); thermal expansion of the co-crystal (Figure S5) (PDF)

## References

[ref1] Hörst S. M. Titan’s Atmosphere and Climate. J. Geophys. Res.: planets 2017, 122 (3), 432–482. 10.1002/2016JE005240.

[ref2] Nixon C. A. The Composition and Chemistry of Titan’s Atmosphere 2024, 8 (3), 406–456. 10.1021/acsearthspacechem.2c00041. PMC10961852 38533193

[ref3] Anderson C. M.; Samuelson R. E.; Nna-Mvondo D. Organic Ices in Titan’s Stratosphere. Space Sci. Rev. 2018, 214 (8), 1–36. 10.1007/s11214-018-0559-5.

[ref4] Barth E. L.; Toon O. B. Ethane, and Mixed Clouds in Titan’s Atmosphere: Properties Derived from Microphysical Modeling. Icarus 2006, 182 (1), 230–250. 10.1016/j.icarus.2005.12.017.

[ref5] Yu X.; Hörst S. M.; He C.; Mcguiggan P. Single Particle Triboelectrification of Titan Sand Analogs. Earth Planet. Sci. Lett. 2020, 530, 115996 10.1016/j.epsl.2019.115996.

[ref6] Tomasko M. G.; Archinal B.; Becker T.; Bézard B.; Bushroe M.; Combes M.; Cook D.; Coustenis A.; De Bergh C.; Dafoe L. E.; et al. Winds and Haze during the Huygens Probe’s Descent to Titan’s Surface. Nature 2005, 438 (7069), 765–778. 10.1038/nature04126. 16319829

[ref7] Cable M. L.; Vu T. H.; Malaska M. J.; Maynard-Casely H. E.; Choukroun M.; Hodyss R. Properties and Behavior of the Acetonitrile-Acetylene Co-Crystal under Titan Surface Conditions. ACS Earth Space Chem. 2020, 4 (8), 1375–1385. 10.1021/acsearthspacechem.0c00129.

[ref8] Vu T. H.; Cable M. L.; Choukroun M.; Hodyss R.; Beauchamp P. Formation of a New Benzene-Ethane Co-Crystalline Structure under Cryogenic Conditions. J. Phys. Chem. A 2014, 118 (23), 4087–4094. 10.1021/jp501698j. 24809894

[ref9] Czaplinski E.; Yu X.; Dzurilla K.; Chevrier V. Experimental Investigation of the Acetylene–Benzene Cocrystal on Titan. Planet. Sci. J. 2020, 1, 3 10.3847/PSJ/abbf57.

[ref10] Francis T. A.; Maynard-Casely H. E.; Cable M. L.; Hodyss R.; Ennis C. Simulation of Cocrystal Formation in Planetary Atmospheres: The C6H6: C2H2 Cocrystal Produced by Gas Deposition. J. Phys. Chem. A 2023, 127 (10), 2322–2335. 10.1021/acs.jpca.2c08791. 36790472

[ref11] Maynard-Casely H. E.; Cable M. L.; Malaska M. J.; Vu T. H.; Choukroun M.; Hodyss R. Prospects for Mineralogy on Titan. Am. Mineral 2018, 103 (3), 343–349. 10.2138/am-2018-6259.

[ref12] Czaplinski E. C.; Vu T. H.; Cable M. L.; Choukroun M.; Malaska M. J.; Hodyss R. Experimental Characterization of the Pyridine: Acetylene Co-Crystal and Implications for Titan’s Surface. ACS Earth Space Chem. 2023, 7 (3), 597–608. 10.1021/acsearthspacechem.2c00377. 36960425 PMC10026175

[ref13] Cable M. L.; Runčevski T.; Maynard-Casely H. E.; Vu T. H.; Hodyss R. Titan in a Test Tube: Organic Co-Crystals and Implications for Titan Mineralogy. Acc. Chem. Res. 2021, 54 (15), 3050–3059. 10.1021/acs.accounts.1c00250. 34296607

[ref14] Yanez M. D.; Cable M. L.; Larowe D. E.; Amend J. Microbial Acetylenotrophy for Future Astrobiology Studies of Ocean Worlds AbScicon Ocean worlds near and far, 2022.

[ref15] Neish C.; Malaska M. J.; Sotin C.; Lopes R. M. C.; Nixon C. A.; Affholder A.; Chatain A.; Cockell C.; Farnsworth K. K.; Higgins P. M.; Miller K. E.; Soderlund K. M. Organic Input to Titan’s Subsurface Ocean Through Impact Cratering. Astrobiology 2024, 24 (2), 177–189. 10.1089/ast.2023.0055. 38306187

[ref16] Vuitton V.; Yelle R. V.; Klippenstein S. J.; Hörst S. M.; Lavvas P. Simulating the Density of Organic Species in the Atmosphere of Titan with a Coupled Ion-Neutral Photochemical Model. Icarus 2019, 324, 120–197. 10.1016/j.icarus.2018.06.013.

[ref17] Cordiner M. A.; Palmer M. Y.; Nixon C. A.; Irwin P. G. J.; Teanby N. A.; Charnley S. B.; Mumma M. J.; Kisiel Z.; Serigano J.; Kuan Y. J.; Chuang Y. L.; Wang K. S. Ethyl Cyanide on Titan: Spectroscopic Detection and Mapping Using Alma. Astrophys. J. Lett. 2015, 800 (1), L14 10.1088/2041-8205/800/1/L14.

[ref18] Kisiel Z.; Nixon C. A.; Cordiner M. A.; Thelen A. E.; Charnley S. B. Propionitrile in the Two Lowest Excited Vibrational States in the Laboratory and on Titan. J. Mol. Spectrosc. 2020, 372, 111324 10.1016/j.jms.2020.111324.

[ref19] Lara L. M.; Lellouch E.; López-Moreno J. J.; Rodrigo R. Vertical Distribution of Titan’s Atmospheric Neutral Constituents. J. Geophys. Res.: planets 1996, 101 (E10), 23261–23283. 10.1029/96JE02036.

[ref20] Coustenis A.; Achterberg R. K.; Conrath B. J.; Jennings D. E.; Marten A.; Gautier D.; Nixon C. A.; Flasar F. M.; Teanby N. A.; Bézard B.; Samuelson R. E.; Carlson R. C.; Lellouch E.; Bjoraker G. L.; Romani P. N.; Taylor F. W.; Irwin P. G. J.; Fouchet T.; Hubert A.; Orton G. S.; Kunde V. G.; Vinatier S.; Mondellini J.; Abbas M. M.; Courtin R. The Composition of Titan’s Stratosphere from Cassini/CIRS Mid-Infrared Spectra. Icarus 2007, 189 (1), 35–62. 10.1016/j.icarus.2006.12.022.

[ref21] Waite J. H. Jr; Niemann H.; Yelle R. V.; Kasprzak W. T.; Cravens T. E.; Luhmann J. G.; McNutt R. L.; Ip W.-H.; Gell D.; De L. H. V.; Müller-Wordag I.; Magee B.; Borggren N.; Ledvina S.; Fletcher G.; Walter E.; Miller R.; Scherer S.; Thorpe R.; Xu J.; Block B.; Arnett K. Ion Neutral Mass Spectrometer Results from the First Flyby of Titan. Science 2005, 308 (5724), 982–986. 10.1029/2003JE002180. 15890873

[ref22] Niemann H. B.; Atreya S. K.; Demick J. E.; Gautier D.; Haberman J. A.; Harpold D. N.; Kasprzak W. T.; Lunine J. I.; Owen T. C.; Raulin F. Composition of Titan’s Lower Atmosphere and Simple Surface Volatiles as Measured by the Cassini-Huygens Probe Gas Chromatograph Mass Spectrometer Experiment. J. Geophys. Res. E Planets 2010, 115 (12), E12 10.1029/2010JE003659.

[ref23] Singh S.; Mccord T. B.; Combe J.; Rodriguez S.; Cornet T.; Mouélic S. L.; Clark R. N.; Maltagliati L.; Chevrier V. F. Acetylene on Titan’s Surface. Astrophys. J. 2016, 828 (1), 1–8. 10.3847/0004-637X/828/1/55.

[ref24] McMullan R. K.; Kvick Å.; Popelier P. Structures of Cubic and Orthorhombic Phases of Acetylene by Single-Crystal Neutron Diffraction. Acta Crystallogr., Sect. B 1992, 48 (5), 726–731. 10.1107/S0108768192004774.

[ref25] Yu X.; Yu Y.; Garver J.; Li J.; Hawthorn A.; Sciamma-O’Brien E.; Zhang X.; Barth E. Material Properties of Organic Liquids, Ices, and Hazes on Titan. Astrophys. J., Suppl. Ser. 2023, 266 (2), 30 10.3847/1538-4365/acc6cf.

[ref26] Cable M. L.; Vu T. H.; Maynard-Casely H. E.; Choukroun M.; Hodyss R. The Acetylene-Ammonia Co-Crystal on Titan. ACS Earth Space Chem. 2018, 2 (4), 366–375. 10.1021/acsearthspacechem.7b00135.

[ref27] Fulchignoni M.; Ferri F.; Angrilli F.; Ball A. J.; Bar-Nun A.; Barucci M. A.; Bettanini C.; Bianchini G.; Borucki W.; Colombatti G.; Coradini M.; Coustenis A.; Debei S.; Falkner P.; Fanti G.; Flamini E.; Gaborit V.; Grard R.; Hamelin M.; Harri A. M.; Hathi B.; Jernej I.; Leese M. R.; Lehto A.; Lion Stoppato P. F.; López-Moreno J. J.; Mäkinen T.; McDonnell J. A. M.; McKay C. P.; Molina-Cuberos G.; Neubauer F. M.; Pirronello V.; Rodrigo R.; Saggin B.; Schwingenschuh K.; Seiff A.; Simões F.; Svedhem H.; Tokano T.; Towner M. C.; Trautner R.; Withers P.; Zarnecki J. C. In Situ Measurements of the Physical Characteristics of Titan’s Environment. Nature 2005, 438 (7069), 785–791. 10.1038/nature04314. 16319827

[ref28] Jasper A. W.; Klippenstein S. J.; Harding L. B. Secondary Kinetics of Methanol Decomposition: Theoretical Rate Coefficients for ^3^ CH _2_ + OH, ^3^ CH _2_ + ^3^ CH _2_, and ^3^ CH _2_ + CH _3_. J. Phys. Chem. A 2007, 111 (35), 8699–8707. 10.1021/jp0736950. 17696414

[ref29] Holland D. M. P.; Shaw D. A.; Hayes M. A.; Shpinkova L. G.; Rennie E. E.; Karlsson L.; Baltzer P.; Wannberg B. A. P. Photodissociation and Photoelectron Spectroscopy Study of C2H4 and C2D4. Chem. Phys. 1997, 219 (1), 91–116. 10.1016/S0301-0104(97)00090-6.

[ref30] Mcintosh D. The Physical Properties of Liquid and Solid Acetylene. J. Phys. Chem. 1907, 11 (4), 306–317. 10.1021/j150085a005.

[ref31] Cui J.; Yelle R. V.; Vuitton V.; Waite J. H.; Kasprzak W. T.; Gell D. A.; Niemann H. B.; Müller-Wodarg I. C. F.; Borggren N.; Fletcher G. G.; Patrick E. L.; Raaen E.; Magee B. A. Analysis of Titan’s Neutral Upper Atmosphere from Cassini Ion Neutral Mass Spectrometer Measurements. Icarus 2009, 200 (2), 581–615. 10.1016/j.icarus.2008.12.005.

[ref32] Lavvas P. P.; Coustenis A.; Vardavas I. M. Coupling Photochemistry with Haze Formation in Titan’s Atmosphere, Part I: Model Description. Planet. Space Sci. 2008, 56 (1), 27–66. 10.1016/j.pss.2007.05.026.

[ref33] Lavvas P. P.; Coustenis A.; Vardavas I. M. Coupling Photochemistry with Haze Formation in Titan’s Atmosphere, Part II: Results and Validation with Cassini/Huygens Data. Planet. Space Sci. 2008, 56 (1), 67–99. 10.1016/j.pss.2007.05.027.

[ref34] Loison J. C.; Hébrard E.; Dobrijevic M.; Hickson K. M.; Caralp F.; Hue V.; Gronoff G.; Venot O.; Bénilan Y. The Neutral Photochemistry of Nitriles, Amines and Imines in the Atmosphere of Titan. Icarus 2015, 247, 218–247. 10.1016/j.icarus.2014.09.039.

[ref35] Kanda K.; Nagata T.; Ibuki T. Photodissociation of Some Simple Nitriles in the Extreme Vacuum Ultraviolet Region. Chem. Phys. 1999, 243 (1–2), 89–96. 10.1016/S0301-0104(99)00063-4.

[ref36] Brand H. E. A.; Gu Q.; Kimpton J. A.; Auchettl R.; Ennisb C. Crystal Structure of Propionitrile (CH3CH2CN) Determined Using Synchrotron Powder X-Ray Diffraction. J. Synchrotron Radiat. 2020, 27 (1), 212–216. 10.1107/S1600577519015911. 31868754

[ref37] Willacy K.; Allen M.; Yung Y. A New Astrobiological Model of the Atmosphere of Titan. Astrophys. J. 2016, 829 (2), 79 10.3847/0004-637X/829/2/79.

[ref38] Hodyss R.; Vu T. H.; Choukroun M.; Cable M. L. A Simple Gas Introduction System for Cryogenic Powder X-Ray Diffraction. J. Appl. Crystallogr. 2021, 54, 1268–1270. 10.1107/S1600576721006671.

[ref39] Anderson A.; Andrews B.; Torrie B. H. Raman and Far Infrared Spectra of Crystalline Acetylene, C2H2 and C2D2. J. Raman Spectrosc. 1985, 16 (3), 202–207. 10.1002/jrs.1250160313.

[ref40] Vu T. H.; Hodyss R.; Cable M. L.; Choukroun M. Raman Signatures and Thermal Expansivity of Acetylene Clathrate Hydrate. J. Phys. Chem. A 2019, 123 (32), 7051–7056. 10.1021/acs.jpca.9b04426. 31310533 PMC6697187

[ref41] Wurrey C. J.; Bucy W. E.; Durig J. R. Vibrational Spectra and Normal Coordinate Analysis of Ethyl Cyanides. J. Phys. Chem. 1976, 80 (11), 1129–1136. 10.1021/j100552a003.

[ref42] Crowder G. A. Vibrational Assignment for Propionitrile. Spectrochim. Acta, Part A 1986, 42 (10), 1229–1231. 10.1016/0584-8539(86)80080-0.

[ref43] Niemann H. B.; Atreya S. K.; Bauer S. J.; Carignan G. R.; Demick J. E.; Frost R. L.; Gautier D.; Haberman J. A.; Harpold D. N.; Hunten D. M.; Israel G.; Lunine J. I.; Kasprzak W. T.; Owen T. C.; Paulkovich M.; Raulin F.; Raaen E.; Way S. H. The Abundances of Constituents of Titan’s Atmosphere from the GCMS Instrument on the Huygens Probe. Nature 2005, 438 (7069), 779–784. 10.1038/nature04122. 16319830

[ref44] Lorenz R. D.; Niemann H. B.; Harpold D. N.; Way S. H.; Zarnecki J. C. Titan’s Damp Ground: Constraints on Titan Surface Thermal Properties from the Temperature Evolution of the Huygens GCMS Inlet. Meteorit. Planet. Sci. 2006, 41 (11), 1705–1714. 10.1111/j.1945-5100.2006.tb00446.x.

[ref45] Turtle E. P.; Perry J. E.; McEwen A. S.; DelGenio A. D.; Barbara J.; West R. A.; Dawson D. D.; Porco C. C. Cassini Imaging of Titan’s High-latitude Lakes, Clouds, and South-polar Surface Changes. Geophys. Res. Lett. 2009, 36 (2), 2008GL036186 10.1029/2008GL036186.

[ref46] Turtle E. P.; Genio A. D. D.; Barbara J. M.; Perry J. E.; Schaller E. L.; McEwen A. S.; West R. A.; Ray T. L. Seasonal Changes in Titan’s Meteorology: SEASONAL CHANGES IN TITAN’S METEOROLOGY. Geophys. Res. Lett. 2011, 38 (3), n/a–n/a. 10.1029/2010GL046266.

[ref47] Turtle E. P.; Perry J. E.; Hayes A. G.; Lorenz R. D.; Barnes J. W.; McEwen A. S.; West R. A.; Del Genio A. D.; Barbara J. M.; Lunine J. I.; Schaller E. L.; Ray T. L.; Lopes R. M. C.; Stofan E. R. Rapid and Extensive Surface Changes Near Titan’s Equator: Evidence of April Showers. Science 2011, 331 (6023), 1414–1417. 10.1126/science.1201063. 21415347

[ref48] Turtle E. P.; Perry J. E.; Barbara J. M.; Del Genio A. D.; Rodriguez S.; Le Mouélic S.; Sotin C.; Lora J. M.; Faulk S.; Corlies P.; Kelland J.; MacKenzie S. M.; West R. A.; McEwen A. S.; Lunine J. I.; Pitesky J.; Ray T. L.; Roy M. Titan’s Meteorology Over the Cassini Mission: Evidence for Extensive Subsurface Methane Reservoirs. Geophys. Res. Lett. 2018, 45 (11), 5320–5328. 10.1029/2018GL078170.

[ref49] Brown M. E.; Roberts J. E.; Schaller E. L. Clouds on Titan during the Cassini Prime Mission: A Complete Analysis of the VIMS Data. Icarus 2010, 205 (2), 571–580. 10.1016/j.icarus.2009.08.024.

[ref50] Barnes J. W.; Clark R. N.; Sotin C.; Ádámkovics M.; Appéré T.; Rodriguez S.; Soderblom J. M.; Brown R. H.; Buratti B. J.; Baines K. H.; Le Mouélic S.; Nicholson P. D. A TRANSMISSION SPECTRUM OF TITAN’S NORTH POLAR ATMOSPHERE FROM A SPECULAR REFLECTION OF THE SUN. Astrophys. J. 2013, 777 (2), 161 10.1088/0004-637X/777/2/161.

[ref51] Lemmon M. T.; Lorenz R. D.; Smith P. H.; Caldwell J. J. Sub-Tropical Cloud Activity near Titan’s 1995 Equinox. Icarus 2019, 331, 1–14. 10.1016/j.icarus.2019.03.042.

[ref52] Dhingra R. D.; Barnes J. W.; Brown R. H.; Burrati B. J.; Sotin C.; Nicholson P. D.; Baines K. H.; Clark R. N.; Soderblom J. M.; Jauman R.; Rodriguez S.; Mouélic S. L.; Turtle E. P.; Perry J. E.; Cottini V.; Jennings D. E. Observational Evidence for Summer Rainfall at Titan’s North Pole. Geophys. Res. Lett. 2019, 46 (3), 1205–1212. 10.1029/2018GL080943.

[ref53] Yu X.; Yu Y.; Garver J.; Zhang X.; McGuiggan P. The Fate of Simple Organics on Titan’s Surface: A Theoretical Perspective. Geophys. Res. Lett. 2024, 51 (1), 1–10. 10.1029/2023GL106156.

[ref54] Singh S.; Combe J.-P.; Cordier D.; Wagner A.; Chevrier V. F.; McMahon Z. Experimental Determination of Acetylene and Ethylene Solubility in Liquid Methane and Ethane: Implications to Titan’s Surface. Geochim. Cosmochim. Acta. 2017, 208, 86–101. 10.1016/j.gca.2017.03.007.

[ref55] Dubouloz N.; Raulin F.; Lellouch E.; Gautier D. Titan’s Hypothesized Ocean Properties: The Influence of Surface Temperature and Atmospheric Composition Uncertainties. Icarus 1989, 82 (1), 81–96. 10.1016/0019-1035(89)90025-0.

[ref56] Stevenson J. M.; Fouad W. A.; Shalloway D.; Usher D.; Lunine J.; Chapman W. G.; Clancy P. Solvation of Nitrogen Compounds in Titan’s Seas, Precipitates, and Atmosphere. Icarus 2015, 256, 1–12. 10.1016/j.icarus.2015.04.019.

[ref57] Cornet T.; Cordier D.; Bahers T. L.; Bourgeois O.; Fleurant C.; Mouélic S. L.; Altobelli N. Dissolution on Titan and on Earth: Toward the Age of Titan’s Karstic Landscapes. J. Geophys. Res.: planets 2015, 120 (6), 1044–1074. 10.1002/2014JE004738.

[ref58] Malaska M. J.; Hodyss R.; Lunine J. I.; Hayes A. G.; Hofgartner J. D.; Hollyday G.; Lorenz R. D. Laboratory Measurements of Nitrogen Dissolution in Titan Lake Fluids. Icarus 2017, 289, 94–105. 10.1016/j.icarus.2017.01.033.

[ref59] Cable M. L.; Vu T. H.; Malaska M. J.; Maynard-Casely H. E.; Choukroun M.; Hodyss R. A Co-Crystal between Acetylene and Butane: A Potentially Ubiquitous Molecular Mineral on Titan. ACS Earth Space Chem. 2019, 3 (12), 2808–2815. 10.1021/acsearthspacechem.9b00275.

[ref60] Le Gall A.; Malaska M. J.; Lorenz R. D.; Janssen M. A.; Tokano T.; Hayes A. G.; Mastrogiuseppe M.; Lunine J. I.; Veyssière G.; Encrenaz P.; Karatekin O. Composition Change Seasonal Bathymetry of Ligeia Mare Titan, Derived from Its Microwave Thermal Emission: Microwave Radiometry of Ligeia Mare. J. Geophys. Res.: planets 2016, 121 (2), 233–251. 10.1002/2015JE004920.

[ref61] Cordier D.; Cornet T.; Barnes J. W.; MacKenzie S. M.; Le Bahers T.; Nna-Mvondo D.; Rannou P.; Ferreira A. G. Structure of Titan’s Evaporites. Icarus 2016, 270, 41–56. 10.1016/j.icarus.2015.12.034.

[ref62] Ennis C.; Cable M. L.; Hodyss R.; Maynard-Casely H. E. Mixed Hydrocarbon and Cyanide Ice Compositions for Titan’s Atmospheric Aerosols: A Ternary-Phase Co-Crystal Predicted by Density Functional Theory. ACS Earth Space Chem. 2020, 4 (7), 1195–1200. 10.1021/acsearthspacechem.0c00130.

[ref63] Cable M. L.; Vu T. H.; Hodyss R.; Choukroun M.; Malaska M. J.; Beauchamp P. Experimental Determination of the Kinetics of Formation of the Benzene-Ethane Co-Crystal and Implications for Titan. Geophys. Res. Lett. 2014, 41 (15), 5396–5401. 10.1002/2014GL060531.

[ref64] Blake D.; Vaniman D.; Achilles C.; Anderson R.; Bish D.; Bristow T.; Chen C.; Chipera S.; Crisp J.; Des Marais D.; Downs R. T.; Farmer J.; Feldman S.; Fonda M.; Gailhanou M.; Ma H.; Ming D. W.; Morris R. V.; Sarrazin P.; Stolper E.; Treiman A.; Yen A. Characterization and Calibration of the CheMin Mineralogical Instrument on Mars Science Laboratory. Space Sci. Rev. 2012, 170 (1–4), 341–399. 10.1007/s11214-012-9905-1.

[ref65] Tice M. M.; Hurowitz J. A.; Allwood A. C.; Jones M. W. M.; Orenstein B. J.; Davidoff S.; Wright A. P.; Pedersen D. A. K.; Henneke J.; Tosca N. J.; Moore K. R.; Clark B. C.; McLennan S. M.; Flannery D. T.; Steele A.; Brown A. J.; Zorzano M.-P.; Hickman-Lewis K.; Liu Y.; VanBommel S. J.; Schmidt M. E.; Kizovski T. V.; Treiman A. H.; O’Neil L.; Fairén A. G.; Shuster D. L.; Gupta S. The PIXL Team. Alteration History of Séítah Formation Rocks Inferred by PIXL X-Ray Fluorescence, x-Ray Diffraction, and Multispectral Imaging on Mars. Sci. Adv. 2022, 8 (47), eabp9084 10.1126/sciadv.abp9084. 36417516 PMC9683721

[ref66] Wiens R. C.; Maurice S.; Barraclough B.; Saccoccio M.; Barkley W. C.; Bell J. F.; Bender S.; Bernardin J.; Blaney D.; Blank J.; Bouyé M.; Bridges N.; Bultman N.; Caïs P.; Clanton R. C.; Clark B.; Clegg S.; Cousin A.; Cremers D.; Cros A.; Deflores L.; Delapp D.; Dingler R.; D’Uston C.; Darby Dyar M.; Elliott T.; Enemark D.; Fabre C.; Flores M.; Forni O.; Gasnault O.; Hale T.; Hays C.; Herkenhoff K.; Kan E.; Kirkland L.; Kouach D.; Landis D.; Langevin Y.; Lanza N.; Larocca F.; Lasue J.; Latino J.; Limonadi D.; Lindensmith C.; Little C.; Mangold N.; Manhes G.; Mauchien P.; McKay C.; Miller E.; Mooney J.; Morris R. V.; Morrison L.; Nelson T.; Newsom H.; Ollila A.; Ott M.; Pares L.; Perez R.; Poitrasson F.; Provost C.; Reiter J. W.; Roberts T.; Romero F.; Sautter V.; Salazar S.; Simmonds J. J.; Stiglich R.; Storms S.; Striebig N.; Thocaven J. J.; Trujillo T.; Ulibarri M.; Vaniman D.; Warner N.; Waterbury R.; Whitaker R.; Witt J.; Wong-Swanson B. The ChemCam Instrument Suite on the Mars Science Laboratory (MSL) Rover: Body Unit and Combined System Tests. Space Sci. Rev. 2012, 170 (1–4), 167–227. 10.1007/s11214-012-9902-4.

[ref67] Bhartia R.; Beegle L. W.; DeFlores L.; Abbey W.; Razzell Hollis J.; Uckert K.; Monacelli B.; Edgett K. S.; Kennedy M. R.; Sylvia M., ; Perseverance’s Scanning Habitable Environments with Raman and Luminescence for Organics and Chemicals (SHERLOC) Investigation; The Author(s), 2021; Vol. 217. 10.1007/s11214-021-00812-z.

[ref68] Akob D. M.; Sutton J. M.; Fierst J. L.; Haase K. B.; Baesman S.; Luther G. W.; Miller L. G.; Oremland R. S. Acetylenotrophy: A Hidden but Ubiquitous Microbial Metabolism?. FEMS Microbiol. Ecol. 2018, 94 (8), 1–14. 10.1093/femsec/fiy103. PMC7190893 29933435

[ref69] Yanez M. D.; LaRowe D. E.; Cable M. L.; Amend J. P. Energy Yields for Acetylenotrophy on Enceladus and Titan. Icarus 2024, 411 (December 2023), 115969 10.1016/j.icarus.2024.115969.

[ref70] Kalousová K.; Wakita S.; Sotin C.; Neish C. D.; Soderblom J. M.; Souček O.; Johnson B. C. Evolution of Impact Melt Pools on Titan. J. Geophys. Res.: planets 2024, 129 (3), e2023JE008107 10.1029/2023JE008107.

[ref71] Hines A.; Cable M.; Hodyss R.; Clayborne A. Replicating Titan Surface Interactions of Binary and Ternary Organic Mixtures in the Lab, 2023.

[ref72] Thakur A. C.; Remsing R. C. Molecular Structure, Dynamics, and Vibrational Spectroscopy of the Acetylene: Ammonia (1: 1) Plastic Co-Crystal at Titan Conditions. ACS Earth Space Chem. 2023, 7, 479–489. 10.1021/acsearthspacechem.2c00327.

