# Supplementary material for: Formation and Stability of the Propionitrile:Acetylene Co-Crystal Under Titan-Relevant Conditions
Source: ACS Earth Space Chem. 2025 Jan 28;9(2):253–64. doi: 10.1021/acsearthspacechem.4c00262 (PMC11849028; doi:10.1021/acsearthspacechem.4c00262)
Supplement: Supplementary file 1 — sp4c00262_si_001.pdf [file sp4c00262_si_001.pdf]

Formation and Stability of the Propionitrile:Acetylene Co-Crystal Under Titan-Relevant Conditions

Ellen C. Czaplinski[1], Tuan H. Vu[1], Helen Maynard-Casely[2], Courtney Ennis[3,4], Morgan L. Cable[1], Michael J. Malaska[1], Robert Hodyss[1]*

[1]NASA Jet Propulsion Laboratory, California Institute of Technology, Pasadena, California 91109, United States

[2]Australian Nuclear Science and Technology Organisation, NSW 2232, Australia

[3]Department of Chemistry, University of Otago, Dunedin 9054, New Zealand

[4]MacDiarmid Institute for Advanced Materials and Nanotechnology, Wellington 6140, New Zealand

KEYWORDS: co-crystalline, nitrile, hydrocarbon, Titan, Raman spectroscopy, X-ray diffraction, molecular mineral, labyrinth terrain

Supporting Information

7 pages, 1 table, 5 figures

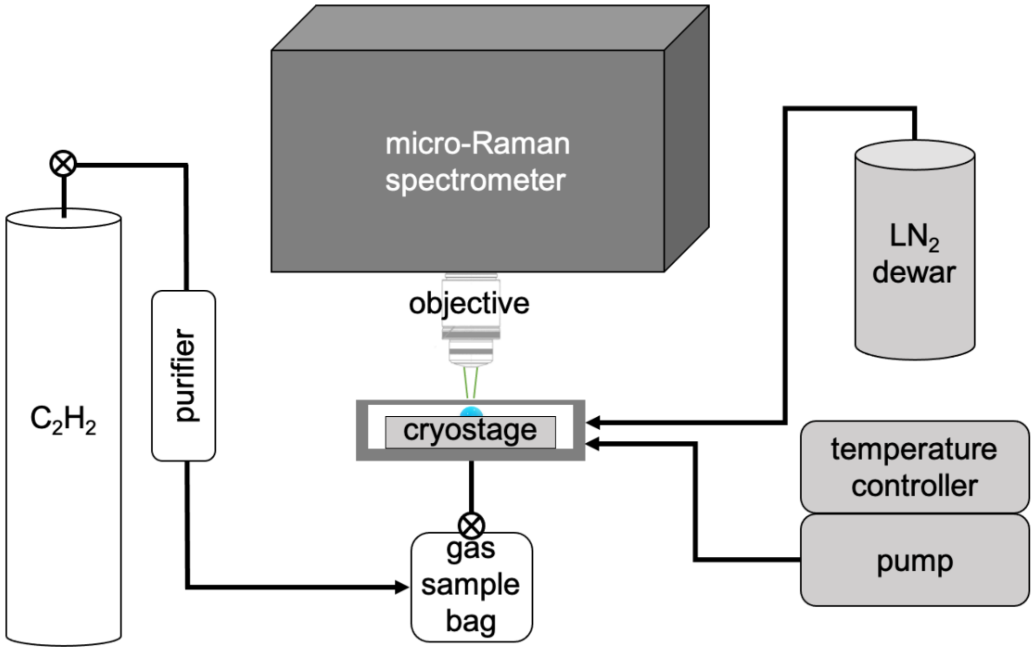

Figure S1. A schematic diagram of the micro-Raman experimental setup. Note the aliquot of liquid propionitrile (denoted by blue half-sphere within the cryostage) that is deposited directly onto the slide within the cryostage. Figure is from Czaplinski et al. (2023).

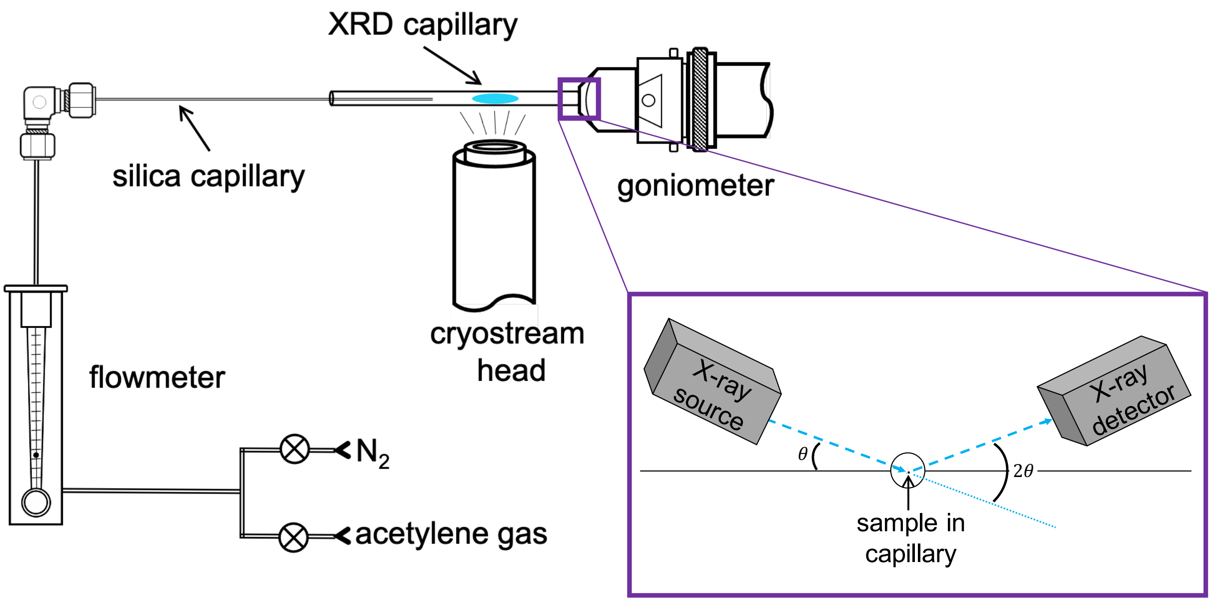

Figure S2. A schematic diagram of the X-ray diffractometer (XRD) setup depicting the custom-built gas introduction system. Bottom right: a front-facing view of the XRD showing the relationship between the X-ray source and the X-ray detector with regard to the sample. Figure is from Czaplinski et al. (2023).

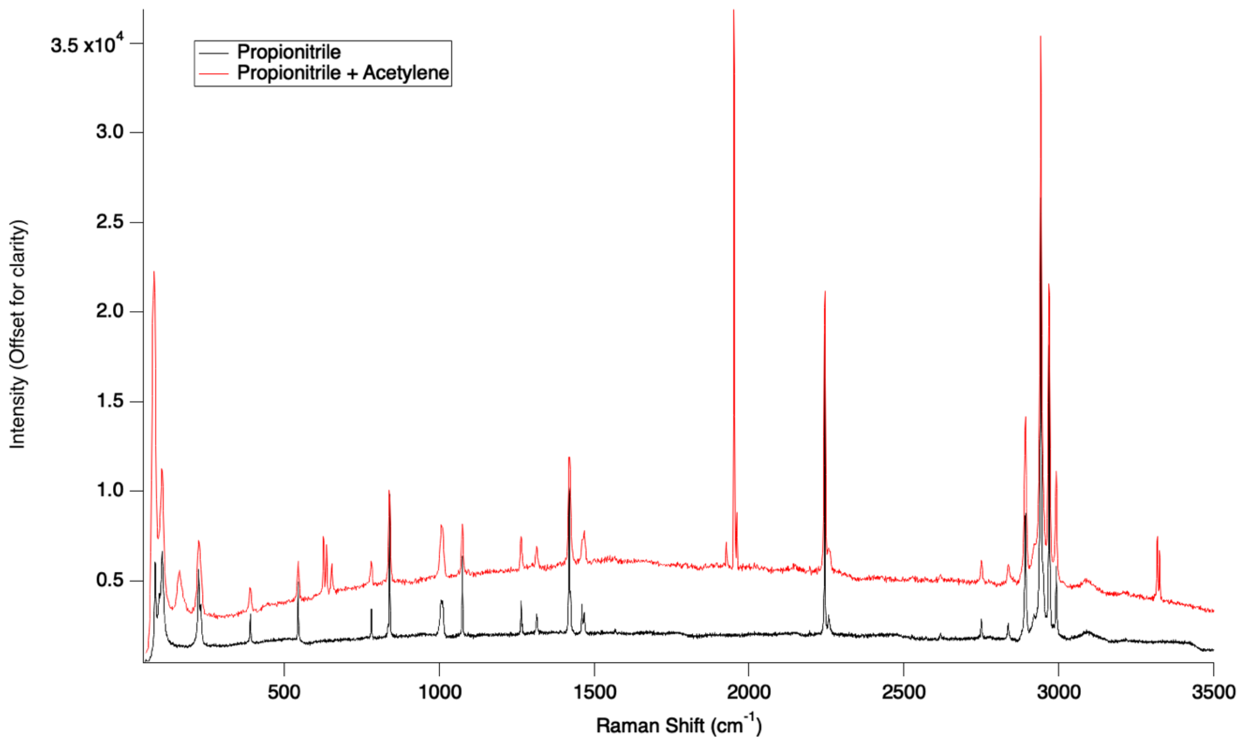

Figure S3. Spectra of pure propionitrile (black) compared to propionitrile after acetylene condensation (red). This combined spectrum in particular focused directly on one of the relatively dark acetylene crystals referenced in the main text (e.g., Figure 2).

Thermal Expansion of Co-Crystal

Table S1. Refined Lattice Constants and Unit Cell Volumes of the Propionitrile:acetylene Co-Crystal from 90−140 K, as Obtained from the Pawley refinement of the Temperature Series Data

Temperature (K)	*a* (Å)	*b* (Å)	*c* (Å)	*β* (deg)	volume (Å^3)
90	8.85(5)	9.816(5)	7.367(7)	116.492(3)	573.1(6)
100	8.88(7)	9.816(7)	7.386(9)	116.442(5)	576.4(9)
110	8.90(1)	9.82(1)	7.409(8)	116.4(1)	579.(1)
120	8.96(1)	9.82(2)	7.468(8)	116.5(1)	587.(2)
130	8.96(1)	9.82(2)	7.47(1)	116.5(1)	588.(2)
140	8.96(1)	9.82(2)	7.47(1)	116.5(1)	588.(2)

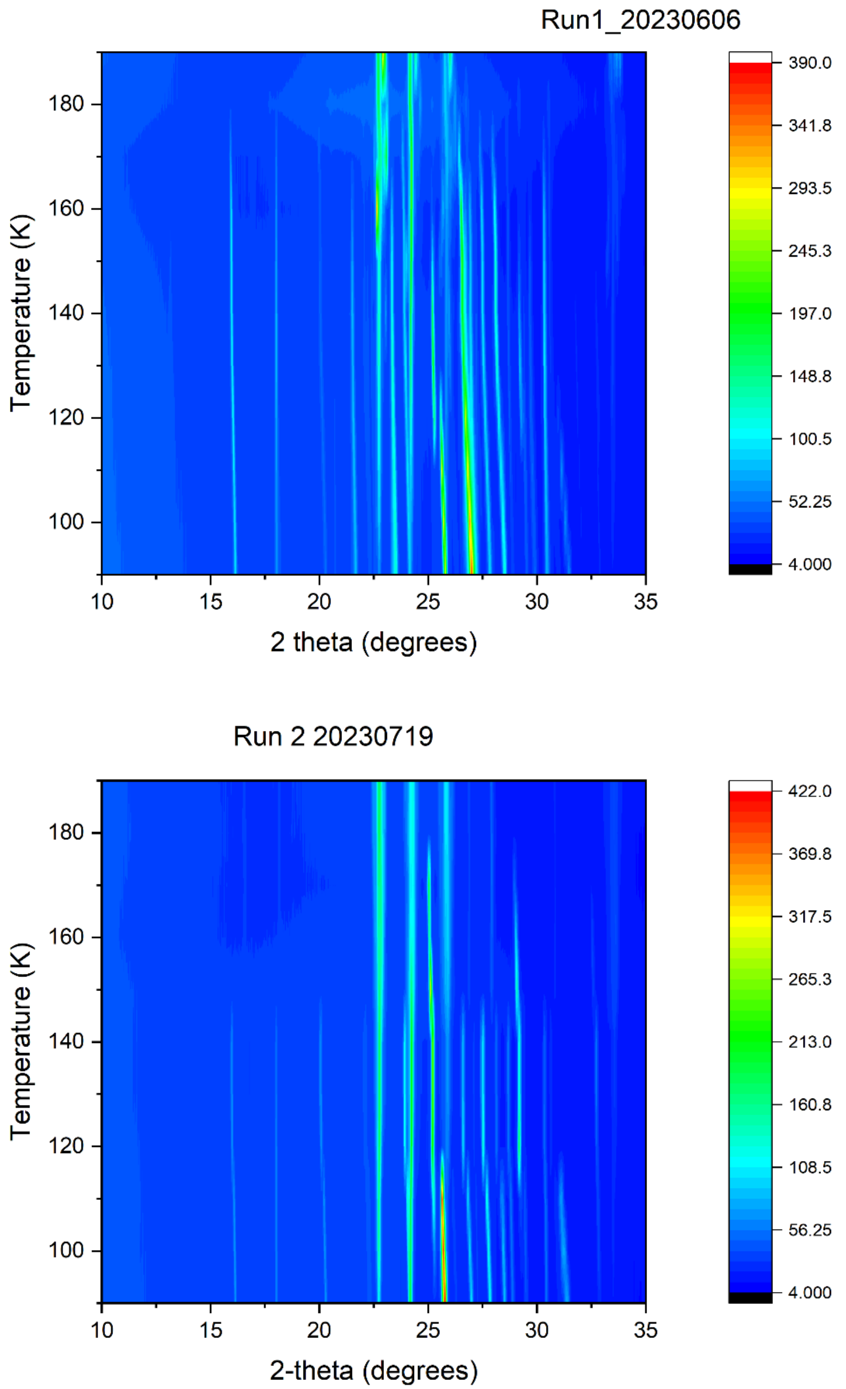

Figure S4. Thermodiffractogram of the co-crystal comparing two separate XRD experiments. Run 1 (top panel) shows the co-crystal peaks persist up to ~180 K, while Run 2 (bottom panel) shows the co-crystal peaks persist up to ~150 K. This could be due to differing concentrations of acetylene between the two experiments, as a result of the experimental setup.

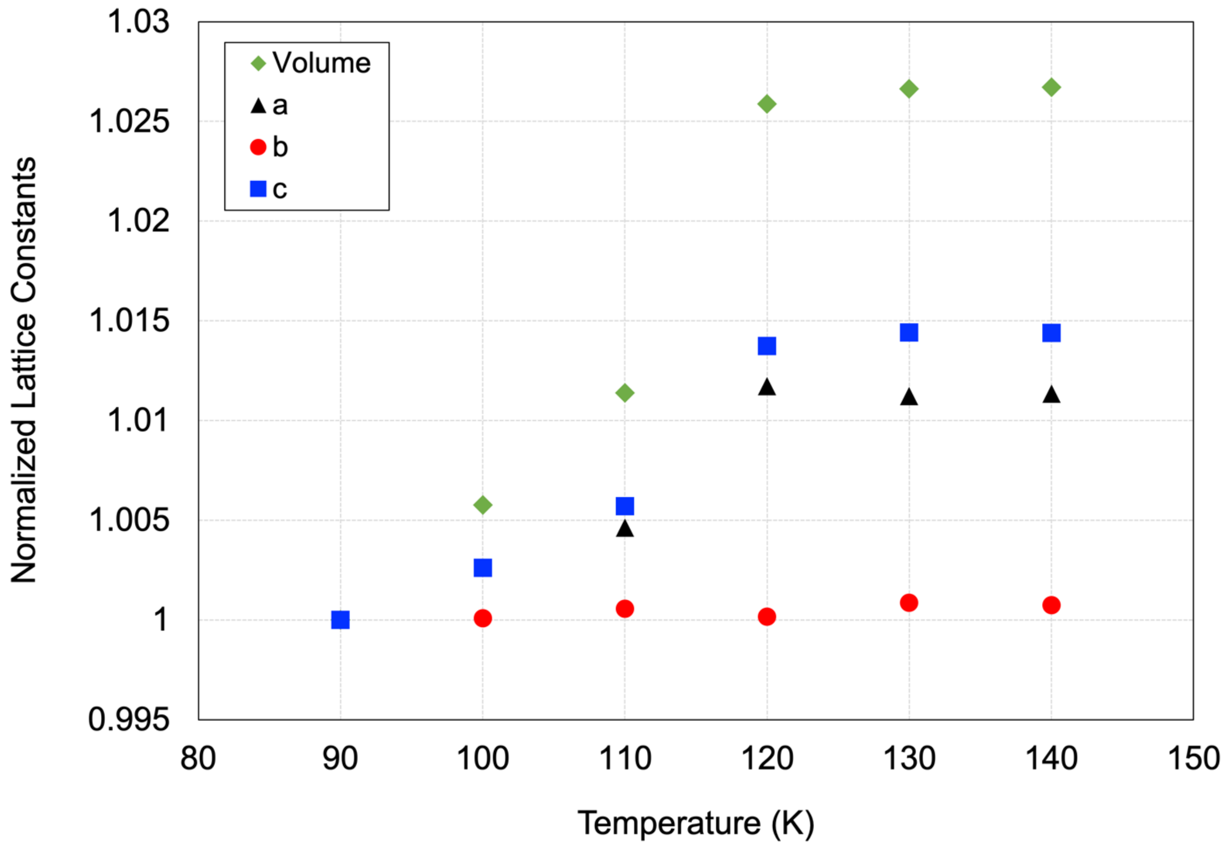

Figure S5. Thermal expansion of the propionitrile:acetylene co-crystal lattice parameters (a, b, c) and unit cell volume as a function of temperature. Note that we only show thermal expansion data from 90-140 K, as an inferred phase transition of propionitrile may exist between 150 K and 170 K -- near the melting point of propionitrile.
